# Robotic mitral valve repair surgery: where do we go from here?

**DOI:** 10.3389/fcvm.2023.1156495

**Published:** 2023-05-24

**Authors:** Anton Tomšič, Meindert Palmen

**Affiliations:** Department of Cardiothoracic Surgery, Leiden University Medical Centre, Leiden, Netherlands

**Keywords:** mitral vale, heart surgery, mitral valve repair, Mitral valve prolaps, robotic surgery

## Abstract

Surgical mitral valve repair through median sternotomy has long presented the treatment of choice for degenerative mitral valve disease. In recent decades, minimal invasive surgical techniques have been developed and are now gaining widespread popularity. Robotic cardiac surgery presents an emerging field, initially adopted only by selected centres, mostly in the United States. In recent years, the number of centers interested in robotic mitral valve surgery has grown with an increasing adoption in Europe as well. Increasing interest and surgical experience gained are stimulating further developments in the field and the full potential of robotic mitral valve surgery remains to be developed.

## Introduction

In Western countries, degenerative mitral valve disease presents the most common indication for surgical mitral valve intervention. In recent decades, we have witnessed a transition from full median sternotomy to less invasive cardiac surgery techniques, driven by a desire to reduce surgical trauma, improve cosmesis and stimulate swifter patient recovery. The transition from median sternotomy to lateral thoracotomy approach occurred already in the 1990s and was pioneered by the groups of Carpentier and Chitwood (1, 2). Simultaneously, thoracoscopic and robot assisted approaches were developed and introduced in regular clinical practice. Ongoing discussion on the quality of valve repair in a minimal invasive setting, when compared to median sternotomy, results from a lack of properly designed comparative studies. Nevertheless, high repair rates combined with low perioperative complication rates and good repair durability have been established in dedicated centres and robotic mitral valve surgery is nowadays a rapid developing field that is increasingly being adopted by dedicated minimal invasive centres worldwide (3).

## The evidence supporting robotic mitral valve surgery

A recent systematic review and meta-analysis including 14 studies and a total of 6,341 patients of whom 2,804 underwent robotic mitral valve surgery and 3,537 patients underwent mitral valve surgery through median sternotomy compared the early results of surgery between both groups (4). Degenerative mitral valve disease was the most common pathology (94.6% in the robotic surgery and 90.5% in the sternotomy group) and, interestingly, the mitral valve repair rate was higher in the robotic surgery group (93.8% vs. 71.0%). This is likely a reflection of increased valve repair complexity and not related to the type of surgical approach. There were no statistically significant differences in the rate of cerebrovascular accidents or reoperations for bleeding. While aortic cross-clamp and cardiopulmonary bypass times were longer in the robotic surgery group, the frequency of postoperative renal insufficiency was lower in this group. Interestingly, there was no statistically significant difference in the rate of postoperative atrial fibrillation or length of mechanical ventilation. On the other hand, robotic surgery was related to shorter intensive care unit and total hospital length of stay.

While the benefits and drawbacks of standard or minimal invasive mitral valve surgery are often discussed in terms of safety and efficacy, patient satisfaction is usually related to other treatment aspects. These include postoperative pain and recovery, cosmetic result and time to return to normal activity. Mitral valve surgery has a known benefit on the physical and emotional well-being of patients suffering from severe mitral regurgitation (5). In a head-to-head comparison between standard and robotic mitral valve surgery, both treatment approaches were effective at improving quality of life with little difference at 2 years after surgery (6). However, the improvement in the quality of life is faster with robotic surgery with prompter return to work and normal daily activity (33 days for robotic repair vs. 54 days for open repair). This early benefit is likely to have a substantial effect on patient satisfaction and significantly reduce the stress related to the operation.

The cost-effectiveness aspect of robotic mitral valve surgery has been studied many times and remains to date controversial. Mihajlevic et al. from the Cleveland Clinic have compared the cost of robotic mitral valve surgery to the costs of either complete sternotomy, partial sternotomy or anterolateral thoracotomy (7). The authors assessed the total costs of implementing a robotic mitral valve surgery program, including the capital investment and fixed yearly maintenance costs. For robotic surgery, the higher technical operative, capital investment and maintenance costs were largely balanced by lower costs of recovery from surgery. Nevertheless, the costs of robotic surgery remained approximately 15% higher when compared to the alternatives. An important observation is the calculated volume-cost relationship of robotic surgery, demonstrating a progressive reduction of additional costs with higher yearly case volumes. Similar conclusions have been made by others (8). A more recent study by Cohan et al. reported no difference in the costs of robotic surgery when compared to median sternotomy (9). Robotic mitral valve surgery was related to shorter hospital admission length and a lower early readmission (within 30 days after surgery) rate. These benefits balanced for the additional costs related to the use of a robotic approach. The cost aspect is of high importance, demanding an optimized and dedicated robotic program to assure cost-effectiveness.

The quality and durability of valve repair following robotic mitral valve surgery has been reported in multiple studies. In 2008, Chitwood et al. studied valve repair durability in 300 patients after robotic mitral valve repair and reported good repair durability, with moderate or greater recurrent regurgitation present in 7.5% of patients at a mean follow-up of 815 ± 459 days (10). However, larger studies with high quality clinical and echocardiographic follow-up at long-term remain scarce (Table 1). Roach et al. studied the results of robotic mitral valve repair in 850 patients, including 582 (68%) patients with isolated posterior leaflet and 268 (32%) with anterior or bileaflet prolapse (11). At a median echocardiographic follow-up of 1.7 (range 0–15) years, the unadjusted 10-year freedom from >2+ recurrent regurgitation or reintervention was 91% for patients with isolated posterior leaflet prolapse and 83% for anterior or bileaflet prolapse. Growing experience was related to a lower risk of recurrent regurgitation. The short median follow-up time and definition of >2+ regurgitation rather than ≥2+ recurrent regurgitation limit the interpretation of the results presented (12). In another study from the same institution, the authors reported a negative effect of residual mild mitral regurgitation grade on late echocardiographic and clinical outcomes (13). Intraoperative repair revision did not result in increased postoperative complications. As expected, these results are in concordance with the lessons learned from mitral valve surgery through median sternotomy (14). At present, the evidence supporting long-term durability of robotic mitral valve repair seems weak and future high quality studies with detailed clinical and echocardiographic follow-up are needed.

**Table 1:** An overview of medium sized studies (>200 patients), reporting long-term follow-up (≥10 years clinical and echocardiographic) of robotic mitral valve repair.

	Country	Number of patients	Study inclusion period	Conversion	Early reoperation for re-valve	Early mortality	Follow-up	Late reoperation	Freedom from reoperation	Echo follow-up	Rec MR	Freedom from rec MR
Aphram et al., 2022	Belgium	278	2012–2022	6 (2.2)	1 (0.4)	0 (0)	N/A	4 (1.4)	98.1 at 10 years	N/A	N/A	67.1% at 10 years
Arghami et al., 2022	USA	843	2008–2019	0 (0)	3 (0.4)	3 (0.4)	3 (IQR 1.1–6) years	15 (1.8)	92.6% at 10 years	6.9 (IQR 0.1–30) mo	53 (6.3)	N/A
Chou et al., 2022	Taiwan	450	2012–2022	0 (0)	0 (0)	1 (0.2)	N/A	9 (2)	98% at 10 years	N/A	N/A^a^	97.6% at 10 years^a^
Roach et al., 2022	USA	850	2005–2022	3 (0.4)	2 (0.2)	1 (0.1)	5.5 (range 0–15) years	17 (2)	N/A	1.7 (range 0–15) years	N/A^b^	91% for isolated posterior and 87% for anterior or bileaflet prolapse at 10 years^b^

Data are presented as *N* (%).

^a^ Defined as more than moderate.

^b^ Defined as greater than 2+.

## Patient selection

Proper patient selection is crucial to secure safety and efficacy of robotic mitral valve repair surgery. It is recommended to perform an electrocardiogram-gated volumetric computed tomography angiography of the chest, abdomen and pelvis before surgery as an addition to the standard screening protocol (15, 16). A recent report form the Cleveland Clinic demonstrated that a conservative screening algorithm will identify a contraindication for robotic intervention in approximately 40% of patients (15). Common contraindications include aortoiliac atherosclerosis, small femoral artery diameter (<7 mm), mitral annular calcification, greater than mild aortic valve regurgitation and/or reduced left ventricular function. Implementation of a conservative screening algorithm resulted in high safety of the robotic surgical intervention with low perioperative stroke and high valve repair rates. The pioneering experience of the Cleveland Clinic presents a reasonable real-world scenario, reflecting the non-exclusive nature of either minimally invasive or open surgical mitral valve procedures. Taking into account the excellent safety and efficacy profile of mitral valve surgery in general, the type of surgical approach should not jeopardize the primary goals of mitral valve surgery. However, these contraindications are only a guideline and center-specific protocols combined with growing experience will allow for further expansion of the profile of patients deemed appropriate for minimal invasive surgery. There are various ways to tackle these limitation. As example, alternative cannulation solutions (bilateral groin cannulation or axillary artery canulation) can be used when the femoral artery are of small diameter or atherosclerotic.

## Robotic versus port-access mitral valve repair

**Figure 1:**
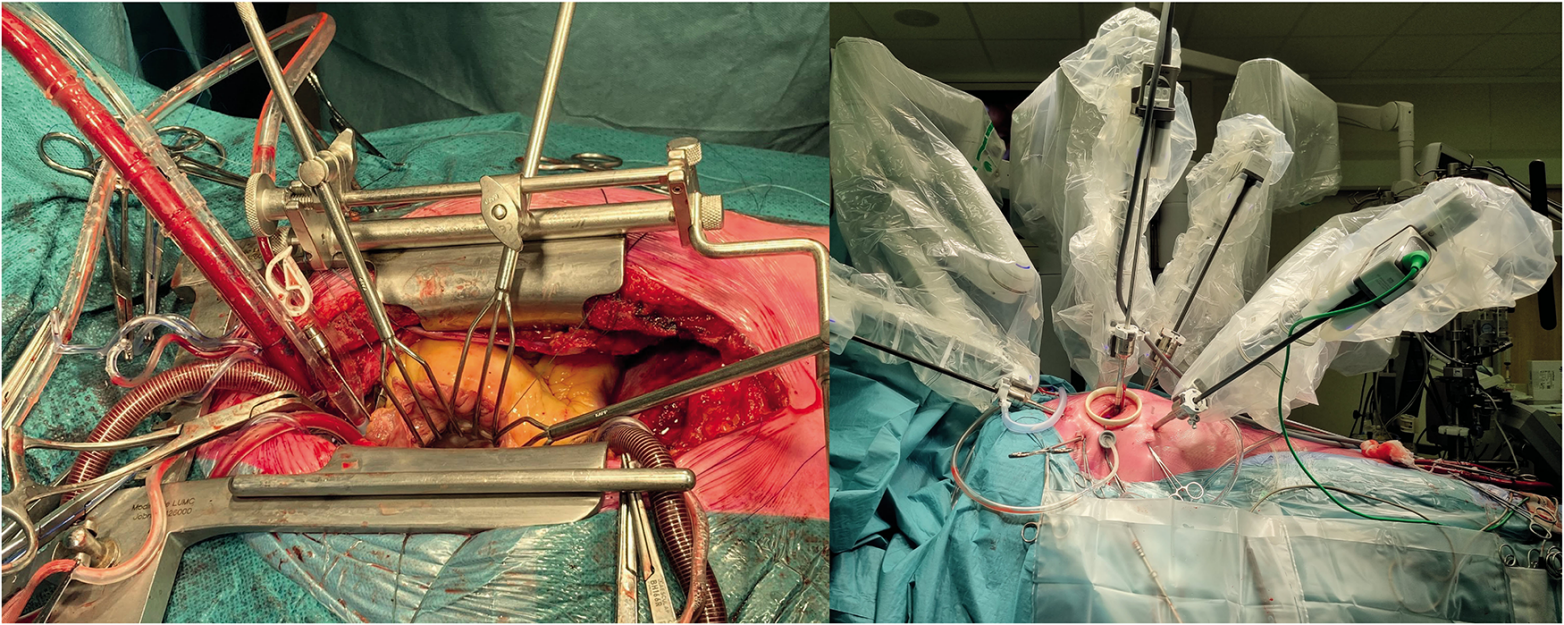
Surgical mitral valve repair through a median sternotomy (left) and robotic lateral mini-thoracotomy approach (right). The lateral mini-thoracotomy approach allows for a less invasive approach without bone fractures.

Various types of minimal invasive mitral valve surgery have been established. Both robotic and port-access mitral valve surgery aim to minimize surgical trauma without jeopardizing treatment effectiveness. In many centres practicing mitral valve repair, median sternotomy remains the gold standard treatment approach. It is performed through midline incision, approximately 15 cm in length, and the same approach is used for cannulation as well as performing surgical repair (Figure 1). The approach offers the benefits of excellent exposure of the heart and mitral valve and high safety profile as it allows the surgeon to react in various ways to any unexpected complications. Median sternotomy is, however, related to prolonged recovery and postoperative restrictions to prevent sternal wound healing problems. Sternal wound infection is a dreaded complication of median sternotomy but this complications is relatively rare in patients with degenerative mitral valve disease. Minimal invasive mitral valve surgery, either endoscopic or robot-assisted, refers most often to the omittance of median sternotomy as the standard surgical access. Lateral minithoracotomy approach, approximately 5 cm in length, with additional work port incisions are used instead and combined with, most often, groin cannulation. The number and size of additional incisions differs in regard to cannulation (percutaneous versus open surgical cannulation) and cross-clamping strategies (external aortic cross-clamping or endoaortic balloon occlusion) applied (17, 18). With minimal invasive surgery, bone fractures and subsequent healing can be avoided, preferably with the use of a soft-tissue retractor without additional rib spreading.

In general, swifter patient recovery following cardiac surgery has been a focus of recent years, with a growing interest in fast track recovery programs and early patient discharge (19). With the development of trans-catheter mitral valve repair technology, the demand for further optimalization of surgical treatment will increase in the future. While trans-catheter techniques are to date unable to reproduce the high quality of surgical intervention, the surgical community should not ignore their presence and, rather, try to utilize the lesson we can learn from them.

The results of both minimal invasive techniques have been compared in only a handful of studies (20). As both techniques use comparable approach and cannulation techniques, little differences in early results are to be expected. The comparison of late results of both treatment approaches is lacking and very few reports with low patient numbers are available (21).

## Discussion

Robotic mitral valve surgery is gaining in popularity and, recently, an increasing adoption of this minimal invasive surgical technique can be seen in the United States and Europe (3, 22). Screening protocols are crucial to prevent unwanted complications. A structured and proctored training program is mandatory to scale the learning curve without jeopardizing results or increasing peri-operative risks for patients. A major limitation to evidence based implementation of robotic mitral valve surgery is the scarcity of studies reporting late results of valve repair. Reports are mainly limited to high volume centres and it remains unproven if the presented results are generalizable to lower volume and less experienced centres. An international registry for robotic mitral valve procedures is currently initiated in Europe and will shed more light on real world outcomes of robotic mitral valve surgery.

The cost aspect of robotic cardiac surgery remains widely debated. Implementation of a robotic program is related to substantial initial investments, ranging from hardware to training. Furthermore, extra procedural costs have to be accounted for because of the use of robotic-associated disposables. However, provided that sufficient volume in terms of operation per year is established, the cost effectiveness of robotic mitral valve surgery seems competitive when compared to standard sternotomy or port-access mitral valve surgery (7, 9). For mitral valve surgery in general, the association between hospital- and surgeon-level volume and 30-day and 1-year mortality rates has previously been identified (23). Other studies have also been able to show a correlation between individual surgeon volume and mitral valve repair and freedom from reoperation rates (24). The concept of Heart Valve Centres is nowadays well established and endorsed by respective guidelines of the European and North American societies (25, 26). In dedicated and specialized teams, minimal invasive mitral valve surgery is becoming one of the pillars of the surgical armamentarium. High procedural volumes and focused clinical interest, both present in Heart Valve Centres, provide an optimal platform for the introduction and development of a successful robotic valve program. Access to a broad arsenal of standard and minimal invasive surgical procedures, as well as trans-catheter procedures, will allow optimal quality of care with a patient-centered approach. Currently, however, less than one-third of isolated mitral valve interventions is performed in a minimal invasive fashion in the United States and only about 10% of isolated mitral valve interventions is performed with robot assistance (27). Taking into account the proportion of patients with no contraindication for minimal invasive surgery, these numbers suggest difficulties with implementation of a minimal invasive program or general reservations with embracement of minimal invasive mitral valve surgery. The general lack of studies with high quality follow-up at long-term might play a role.

A recent nationwide study from the Netherlands suggested, somehow unexpectedly, that minimal invasive, thoracoscopic mitral valve repair might come at a cost of slightly reduced repair and higher reintervention rates in a real-world setting (28). Whether this is related to a lower threshold for valve replacement or other factors is unclear. Regardless of the cause behind these results, minimal invasive mitral valve replacement is inferior to mitral valve repair through median sternotomy. When doubts regarding valve repairability are present, median sternotomy should remain the preferred treatment approach.

When compared to port-access mitral valve surgery, the robotic platform offers benefits in terms of high-definition 3-dimensional visualization and magnification, enhanced surgical dexterity and excellent precision enabled by robotic instruments. Whether this is related to an improvement of clinical and echocardiographic outcomes remains unknown. Based on the evidence available, both approaches offer comparable early results (29, 30). Future high quality clinical studies are needed to explore the durability of valve repair following minimal invasive surgery. In particular, long-term echocardiographic follow-up remains underreported and insufficiently explored.

Last but not least, future problems arising from the incorporation of the new European Medical Device Regulation need to be discussed. The United States have been a forerunner in the development and implementation of robotic cardiac surgery systems and remain a pioneer in the field. Growing interest in several European centres has allowed this technology to be successfully introduced to the European market as well. Cardiac surgery, however, remains a highly-specialized niche where proper manufacturer support is crucial to secure patient safety. Innovation has always been in the heart of the cardiac surgical community, allowing for rapid development of the field over the course of only a few decades. While regulations are clearly needed to serve the interests of the patients and protect their safety, these regulations should not prevent the use of readily certified innovative treatment options, block innovation and future development of the field, depriving these same patients from the benefits that new technology offers. Robotic cardiac surgery is a young and developing field and recently, among other improvements, a new system allowing introduction of robotic tools through a single port has been introduced (Intuitive Surgical, Inc., Sunnyvale, CA, USA). Robotic cardiac surgery has been introduced in low- and middle-income countries where shorter hospitalization times and prompter return to daily activities are important to tackle the shortage in hospital capacities (31). Moreover, the robotic system offers the, to date, theoretical benefit of telementoring and telesurgery, potentially aiding in the reduction of global surgical disparities. Without innovation, degenerative mitral valve surgery would still be an untreatable disease.

## Future perspective

While robotic mitral valve surgery is growing in popularity in the recent years, the number of centres that adopted this technology and the proportion of patients undergoing robotic mitral valve repair remains limited. A limited availability of robotic platforms supported by the industry, especially in the light of the new European Medical Device Regulations, further hampers expansion of these procedures. A number of limitations and practical considerations are likely also responsible for the slow adoption. The number of centres with sufficient case volume load remains limited, particularly when the proportion of patients planned to undergo mitral valve surgery without contraindication for minimal invasive surgery is considered. Moreover, the evidence supporting superiority of robotic mitral valve surgery, when compared to median sternotomy, remains scarce and limited to retrospective studies, prone for bias. Many experienced surgeons therefore remain reluctant to adopt this new technology (32).

In light of the controversy regarding the reproducibility and introduction of robotic cardiac surgery in general, the desire of the patients to reduce the burden of surgical intervention should be sufficiently appreciated. The burden of medical innovation and hereto related learning curve holds a controversial price. The early pioneering times of cardiac surgery, characterized by lack of treatment alternatives and performance benchmarks, are long gone. Standard surgical procedures, in particular mitral valve surgery through median sternotomy, are safe, reproducible and effective. However, the competition of trans-catheter techniques, albeit less effective and with less durable results, is changing the perspective of clinicians and patients. Minimizing surgical trauma and swift postoperative recovery are unfulfilled goals of the cardiac surgical community that were already in the mind of Alain Carpentier, a profound believer in minimal invasive mitral valve surgery, in year 1983 (33). Robotic and other types of minimal invasive surgery will have to demonstrate clear advantages over median sternotomy in the future to make the dream of the pioneer of mitral valve surgery a reality.
